# Editorial: Hypertension and endocrine pathways: molecular and clinical perspectives

**DOI:** 10.3389/fendo.2026.1994648

**Published:** 2026-09-21

**Authors:** Nicolas F. Renna, Mariela Mercedes Gironacci, Nina Japundžic-Žigon

**Affiliations:** 1 Department of Physiopathology, School of Medicine-Universidad Nacional de Cuyo, Mendoza, Argentina; 2 Department of Cardiology, Hospital Español de Mendoza, Mendoza, Argentina; 3 Universidad de Buenos Aires, Facultad de Farmacia y Bioquímica, and Instituto de Química y Fisicoquímica Biológicas “Prof. Alejandro C. Paladini” (UBA-CONICET), Buenos Aires, Argentina; 4 Institute of Pharmacology, Clinical Pharmacology and Toxicology, Medical Faculty University of Belgrade, Belgrade, Serbia

**Keywords:** aldosterone, cushing, hypertension, insulin resistance, pathway

Hypertension is often classified by blood pressure thresholds, yet its biology extends far beyond hemodynamics. Hormonal signaling, autonomic regulation, metabolism, inflammation, renal handling of sodium, and reproductive physiology interact throughout the course of the disease. The articles in this Research Topic examine these interactions across molecular, clinical, and population levels. Together, they support a view of hypertension as a heterogeneous endocrine-vascular syndrome in which similar blood pressure values may arise from different mechanisms and carry different patterns of target-organ risk.

## Adrenal signaling and aldosterone phenotypes

Several contributions place adrenal biology at the center of mechanism-based hypertension. Long and Azizan review adrenal anatomy, steroidogenesis, and the genetic basis of primary aldosteronism, linking advances in somatic and germline genetics with improved disease classification. Lima Sobrinho and Almeida extend this molecular perspective through the cAMP/PKA signaling pathway, showing how alterations in GNAS, PRKAR1A, phosphodiesterases, and PRKACA connect adrenal steroid excess, vascular remodeling, and distinct hypertensive syndromes. These reviews illustrate why endocrine hypertension cannot be reduced to a short list of secondary causes: intracellular signaling and genetic variation shape both hormone production and vascular response.

This mechanistic continuum is reflected in the clinical studies of aldosterone excess. A data-driven analysis of renin-independent aldosteronism identified three reproducible phenotypes with different degrees of metabolic dysfunction, aldosterone excess, cardiac remodeling, and cardiovascular events (Liu et al). The subgroup with the greatest metabolic disturbance had the highest cardiovascular risk, suggesting that aldosterone biology and metabolic context should be assessed together. A complementary review of subclinical primary aldosteronism describes renin-independent aldosterone secretion as a graded phenotype that may precede overt hypertension and conventional diagnostic thresholds (Akoum et al.). This concept has important implications for screening, although prospective trials are required before early mineralocorticoid receptor blockade can be recommended.

The clinical consequences of fluctuating endocrine activity are illustrated by the case of cyclic Cushing syndrome treated with a continuous block-and-replace strategy (Suchy et al.). Recurrent changes in cortisol secretion paralleled severe instability in blood pressure and glucose control. The case emphasizes the diagnostic limitations of isolated hormonal measurements and the need to interpret endocrine tests within a longitudinal clinical trajectory.

## Metabolic phenotypes and target organ risk

A second group of articles examines the metabolic and inflammatory heterogeneity of hypertension. Integrated targeted and untargeted metabolomics identified abnormalities across the kynurenine, serotonin, and microbial-indole branches of tryptophan metabolism in treatment-naive hypertension (Yue et al.). Although the sample was small, the results provide testable links between immune signaling, the gut microbiome, neurotransmitter pathways, and vascular regulation. At the clinical phenotype level, Zhao et al. propose an insulin resistance-systemic vascular resistance-isolated diastolic hypertension axis. Their framework is particularly relevant to younger and middle-aged adults, in whom elevated diastolic pressure may reflect resistance-vessel dysfunction coupled with metabolic abnormalities.

Simple biomarkers may help translate these mechanisms into risk stratification. Among 2,052 patients with newly diagnosed hypertension, a high triglyceride-glucose index was associated with microalbuminuria after propensity-score adjustment. In a separate ambulatory blood pressure monitoring cohort (Wang et al.), TyG-BMI and C-reactive protein were independently associated with isolated nocturnal hypertension, reinforcing the relation between metabolic stress, inflammation, and circadian blood pressure dysregulation (Kaynak et al.). These observational findings do not establish causality, but they support closer integration of metabolic assessment, albuminuria testing, and out-of-office blood pressure measurement.

Prediction studies in the collection broaden this approach. A multimodal model combining catecholamine-related markers, uric acid, renal indices, and clinical variables showed good discrimination for prevalent hypertensive organ damage in training and validation samples (Wang et al.). A prospective cohort of 12,893 hypertensive adults found a graded association between the LDL-C/HDL-C ratio and first stroke (Yu et al.). Both studies point toward multidimensional risk assessment, but external validation and demonstration of clinical utility remain necessary before such markers alter routine care. The protocol evaluating Yiqi-Shengjin granules added to losartan in patients with hypertension and abnormal glucose metabolism adds an interventional perspective and highlights the need for controlled evaluation of therapies intended to address both blood pressure and metabolic dysfunction (Shi et al.).

## Pregnancy and reproductive vascular health

The Research Topic also shows how endocrine-vascular interactions vary across the life course. In a cohort of 84,298 pregnancies, higher uric acid concentrations from early to mid-pregnancy were associated with subsequent hypertensive disorders of pregnancy, with associations that differed by gestational age and clinical subtype (Zou et al.). A smaller case-control study found that serum uric acid had better diagnostic discrimination for preeclampsia than other renal indices and the cerebroplacental ratio (Chen and Lian). These results support uric acid as an accessible risk marker, while leaving unresolved whether it is a causal mediator or a correlate of placental, renal, and endothelial dysfunction.

A systematic review and meta-analysis of the hypothalamic-neurohypophyseal system in preeclampsia identified elevated copeptin, a surrogate of arginine vasopressin secretion, as a consistent feature of preeclampsia and suggested geographic and genetic variation in the strength of this association (Moreira et al.). Beyond pregnancy, a nationwide cohort of more than 2.5 million women linked hypertension identified in early adulthood with a modest increase in incident uterine fibroids (Kang et al.). The study expands the clinical context of hypertension toward reproductive health, while its observational design requires cautious interpretation of shared endocrine, vascular, and inflammatory pathways.

## Implications and future directions

Across these studies, the common message is that blood pressure is an observable endpoint of multiple interacting systems. Aldosterone secretion may be clinically relevant below traditional thresholds; metabolic dysfunction can modify risk within an endocrine phenotype; circadian and reproductive contexts reveal abnormalities that office measurements may miss; and routinely available biomarkers may help identify early organ involvement. The evidence is nevertheless heterogeneous. Several reports are retrospective or single-center, biomarker thresholds are not standardized, and prediction models require broader validation. Associations should therefore guide hypothesis generation and improved phenotyping rather than immediate causal or therapeutic conclusions.

Future studies should combine standardized hormonal testing, ambulatory blood pressure monitoring, metabolic characterization, and longitudinal assessment of target-organ outcomes. Mechanistically informed trials are needed to determine whether earlier treatment of renin-independent aldosterone excess, insulin resistance, inflammation, or other endocrine pathways improves outcomes beyond conventional blood pressure lowering. By connecting molecular mechanisms with clinically recognizable phenotypes, this Research Topic provides a foundation for a more precise approach to hypertension across different ages and clinical settings.

